# An *EAV-HP* insertion in the promoter region of *SLCO1B3* has pleiotropic effects on chicken liver metabolism based on the transcriptome and proteome analysis

**DOI:** 10.1038/s41598-021-87054-9

**Published:** 2021-04-07

**Authors:** Jianfei Chen, Guoying Hua, Deping Han, Xiaotong Zheng, Xianggui Dong, Shuxiang Wang, Junjiang Long, Zhonghua Zheng, Ailing Wang, Jiankui Wang, Xiaotong Wang, Xuemei Deng

**Affiliations:** 1 Key Laboratory of Animal Genetics, Breeding and Reproduction of the Ministry of Agriculture and Rural Affairs and Beijing Key Laboratory of Animal Genetic Improvement, China Agricultural University, Beijing, 100193 China; 2 College of Veterinary Medicine, China Agricultural University, Beijing, 100193 China; 3 Shandong Longsheng Agriculture and Animal, Husbandry Group Co., Ltd, Linyi, 276000 China; 4 School of Agriculture, Ludong University, Yantai, 264000 China

**Keywords:** Agricultural genetics, Animal breeding, Functional genomics, Gene expression, Gene regulation, Genetic markers, Genomics

## Abstract

Solute carrier organic anion transporter 1B3 (*SLCO1B3*) is an important liver primarily highly expressed gene, its encoded protein (OATP1B3) involved in the transport of multi-specific endogenous and exogenous substances. We previously reported that an *EAV-HP* inserted mutation (IM+) in the 5ʹ flanking region of *SLCO1B3* was the causative mutation of chicken blue eggs, and a further research showed that IM+ significantly reduced the expression of *SLCO1B3* in liver. Herein, we confirmed a cholate response element (IR-1) played an important role in activating *SLCO1B3* and in vitro experiments showed that the activation of IR-1 can be significantly reduced by the *EAV-HP* IM+ . We performed transcriptome and proteomic analysis using the same set of IM+ and IM− liver tissues from Yimeng hens (a Chinese indigenous breed) to study the effect of *SLCO1B3* and OATP1B3 expression reduction on chicken liver function. The results showed that common differential expression pathways were screened out from both transcriptome and proteome, in which fatty acid metabolism and drug metabolism—cytochrome P450 were significantly enriched in the KEGG analysis. The lipid-related metabolism was weakened in IM+ group, which was validated by serum biochemical assay. We unexpectedly found that *EAV-HP* fragment was highly expressed in the liver of the IM+ chickens. We cloned the *EAV-HP* full-length transcript and obtained the complete open reading frame. It is worth noting that there was some immune related differential expressed genes, such as *NFKBIZ*, *NFKBIA*, and *IL1RL1*, which were higher expressed in the IM+ group, which may due to the high expression of *EAV-HP*. Our study showed that *EAV-HP* IM+ reduced the expression of *SLCO1B3* in liver, resulting in the decrease of fatty metabolism and exogenous substance transport capacity. The mutation itself also expressed in the liver and may be involved in the immune process. The mechanism needs further study.

## Introduction

The human solute carrier organic anion transporter family member 1B3 *(SLCO1B3*) gene encodes an important membrane transporter protein, organic anion transporter family member 1B3 (OATP1B3), which is specifically expressed at the basolateral membrane of the liver^1,2^. The OATP family member proteins mediate the sodium-independent transport of many amphipathic organic compounds, including bile salts, eicosanoids, steroids, and thyroid hormones^3^. The OATP1B3 protein has been widely investigated with regard to drug transport and deposition in humans^4–7^, especially paclitaxel and CCK-8^8–10^. *SLCO1B3* polymorphisms in humans are closely related to Rotor syndrome and hyperbilirubinemia, which blocks bile salts from the blood from being transported into the liver^11–14^. Furthermore, OATP1B3 also plays an important role in xenobiotic metabolism, which includes drugs and toxins^15,16^. The substrates transported by OATP1B3 are mostly ligands for nuclear receptors, such as the farnesoid X receptor (FXR), pregnenolone X receptor (PXR), and aryl hydrocarbon receptor (AHR)^17–20^, suggesting that OATP1B3 in the liver could regulate gene expression by xenobiotic-activated transcription factors. As an important endogenous and exogenous transporter protein, chicken OATP1B3 in the liver is also considered to play an important role during metabolism.

In a previous study, we have cloned a partial endogenous retroviral (*EAV-HP*) insertion in the 5*ʹ* flanking region of the *SLCO1B3*, this insertion promoted the ectopic high expression of *SLCO1B3* in shell-gland, and it was found to cause chickens to have blue eggshells^21^. A post-gene mapping study conducted genetic investigation of the *SLCO1B3*^22–24^; however, its function in chickens has not been widely investigated. Recently, an expression profile of *SLCO1B3* was outlined in different tissues between the *EAV-HP* inserted mutation (IM+) and wild type (IM−) hens, and it indicated that chicken *SLCO1B3* also mainly specific expressed in the liver and its expression was significantly decreased in the livers of IM+ chickens^25^. This means that IM+ chickens could be used as an animal model to investigate the function of *SLCO1B3* in liver.

Transcriptome and proteome analysis techniques in recent years have advanced to enable differentially expressed genes and proteins in the same tissues to be profiled. Here, to explore the possible effects of the decreased *SLCO1B3* expression on the expression of other genes and associated biological pathways in the IM+ Yimeng chickens, we have collected chicken livers and performed RNA-seq and utilized tandem mass tag (TMT) techniques, to analyze their transcriptome and proteome profiles. We are trying to reveal that the inserted mutation of *SLCO1B3* leads to the different expression regulation patterns in shell-gland and liver, and also affects its function in these two tissues.

## Results

### Identification of the IR-1 like element in the upstream of *SLCO1B3* in vitro

**Figure 1:**
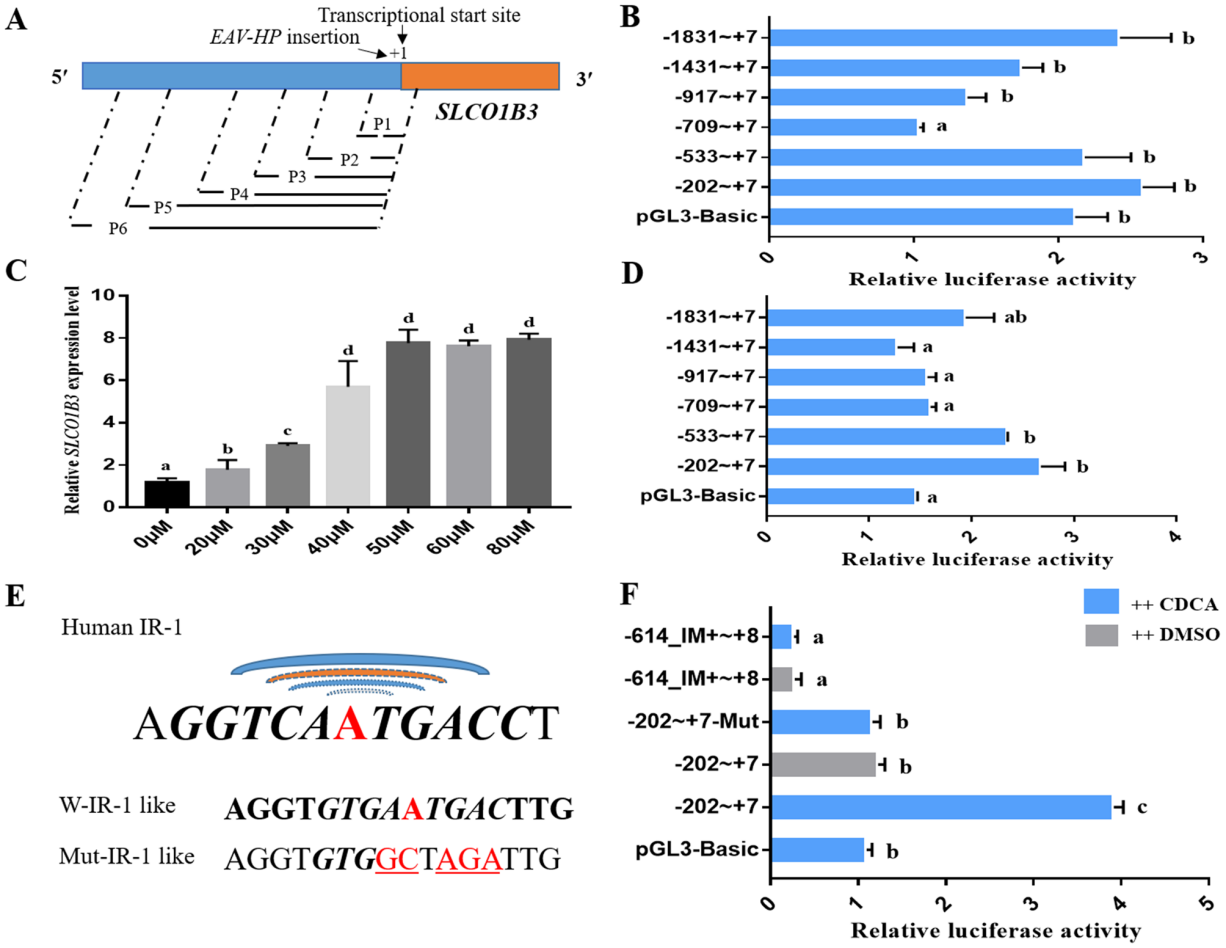
Dual luciferase assays to verify chicken IR-1 like element. (**A**) Schematic diagram of promoter fragments of *SLCO1B3* with different lengths constructed into pGL3.Basic vector. P1: − 202 ~  + 7; P2: − 533 ~  + 7; P3: − 709 ~  + 7; P4: − 917 ~  + 7; P5: − 1431 ~  + 7; P6: − 1831 ~  + 7. (**B**) Relative luciferase activity of serial constructed vectors with insertions of different length 5ʹ flanking regions of *SLCO1B3* after transfection into LMH cells. (**C**) Expression levels of *SLCO1B3* of LMH cells when cultured with different CDCA concentration in the medium. (**D**) Relative luciferase activity of serial constructed vectors with insertions of different length of 5ʹ flanking regions of *SLCO1B3* after transfection into LMH cells with 50 μM CDCA in the complete medium. (**E**) The sequence of human *SLCO1B3* IR-1 element, the predicted chicken IR-1 like element and its mutation type in this assay. Chicken predicted IR-1 element is not similar with the human IR-1 element and it’s located in the 5ʹ flanking region of *SLCO1B3*(-202 ~  + 7). (**F**) Relative luciferase activity of the − 202 ~  + 7 fragment, its predicted IR-1 like mutant constructed vectors (− 202 ~  + 7-Mut) and an IR-1 plus *EAV-HP* insertion fragment vector (− 614_IM+  ~  + 8) after transfection into LMH cells with 50 μM CDCA or DMSO in the compete medium. Different letters indicate significant differences (*P* ≤ 0.05).

In order to identify the upstream regulatory element that activate chicken *SLCO1B3* expression, we constructed luciferase vectors with different length of the 5′ region of *SLCO1B3* to do dual luciferase assays in chicken liver hepatocellular carcinoma (LMH) cells (Fig. 1A). LMH cells have been extensively used as model for ligand-dependent activation of endogenously expressed nuclear receptors^17,26^. We found that in the absence of activator, the region covering ~ 2 Kb upstream of the *SLCO1B3* showed no obvious activation compared with pGL3-Basic vector (Fig. 1B). It has been reported that *SLCO1B3* can be regulated by bile acids in human^17^. We speculate that the regulation of *SLCO1B3* expression also depends on bile acids stimulation. Then, we used different concentrations of chenodeoxycholic acid (CDCA, one of the bile acids) to culture LMH cells. After incubating 36 h with CDCA, *SLCO1B3* expression was detected using quantitative real-time PCR (qPCR). The expression of *SLCO1B3* increased with the increase of CDCA content, and it did not increase after 50uM concentration (Fig. 1C).

The luciferase assays showed that − 202 ~  + 7 bp fragment vector had the highest activities with 50 μM CDCA stimulation (Fig. 1D). In this region, we predicted an inverted hexanucleotide repeat motif (IR-1) like element^25^, which can be stimulated by bile acids. When we mutant the IR-1 like element (Fig. 1E), the expression of dual luciferase report gene decreased (Fig. 1F), the same thing happens when we add partial *EAV-HP* insertion upstream to the IR-1 like element. Though the dual luciferase experiment, we verified the existence of IR-1 like element in the upstream of *SLCO1B3* as activator, and the *EAV-HP* insertion reduced the transcriptional activation of IR-1 like (Fig. 1F).

### Differential expression of the genes screened from RNA-seq and the proteome

Six liver tissue cDNA libraries were established (n = 3 IM+ and n = 3 IM− hens), which represented the case control samples with or without the *EAV-HP* insertion in the promoter of *SLCO1B3*. The RNA-seq generated from 4.25 Gb to 5.78 Gb of clean reads for each library, with an average of 5.24 and 4.70 Gb of paired-end reads for the IM+ and IM− groups, respectively. The clean reads were used for all further analyses. Of the reads in each library, 87.3% to 92.2% were uniquely mapped to the chicken reference genome, and the average mapping rates were 89.45% and 89.33%, for the IM+ and IM− groups, respectively (Table 1).

**Table 1:** Characteristics of the RNA-seq data from 6 chicken liver libraries.

Sample ID	Number of clean bases (Gb)	Clean reads	Q20 (%)	Q30 (%)	Read mapping rate ^a^ (%)
IM+ -1	5.98	57,266,398	96.81	93.58	88.9%
IM+ -2	4.84	47,986,994	95.52	91.13	87.3%
IM+ -3	5.00	49,516,718	96.01	92.48	92.2%
IM– -1	4.26	42,155,298	97.01	93.95	89.6%
IM– -2	4.64	45,932,376	96.97	93.84	88.9%
IM– -3	5.20	51,495,992	96.70	93.37	89.6%

^a^Read mapping ratio, mapped reads/all reads.

After the assembly, 12,802 genes were identified using the RNA-seq analysis, of which 989 were not annotated. We used the annotated genes to select the differentially expressed genes (DEGs). A total of 142 DEGs were selected, using the criteria of a fold change ≥ 2 and a false discovery rate (*P* adjust) ≤ 0.05, and of these, 67 were upregulated and 75 were downregulated. The details of the DEGs are listed in Table S1.

Proteome analysis was performed using the same samples as for the transcriptome. By comparing the reference genome, we identified a total of 3481 proteins. The criteria for selecting differentially expressed proteins (DEPs) were a fold change ≥ 1.2 and a *P* value ≤ 0.05. There were 75 DEPs identified, of which 31 were upregulated and 44 were downregulated. Detailed information of the DEPs is listed in Table S2.

### *SLCO1B3* and OATP1B3 expression levels in the RNA-seq and proteome

**Figure 2:**
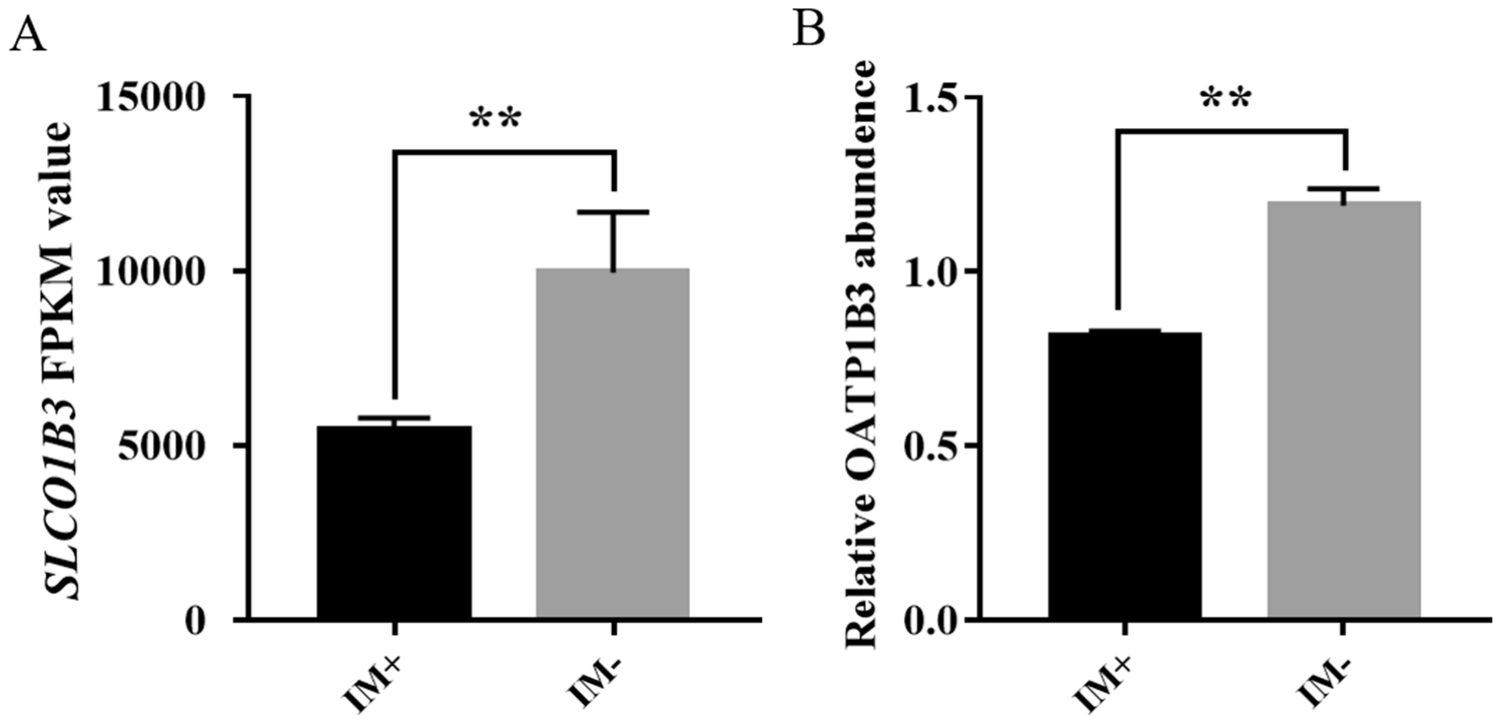
*SLCO1B3* (**A**) and OATP1B3 (**B**) expression in the transcriptome and proteome. The expression of *SLCO1B3* and OATP1B3 of IM+ individuals have about 1/twofold than that in IM− individuals. IM+ chickens can be looked as liver *SLCO1B3* knock-down animal model to do the functional analysis of *SLCO1B3*. **indicates significant differences (*P* ≤ 0.01).

The chickens used in this study were randomly selected from the same population of Yimeng chickens according to the eggshell color. We previously reported different levels of *SLCO1B3* expression in the liver, which were associated with the IM insert mutation in Yimeng chickens^25^. As predicted, RNA-seq analysis revealed that the expression levels of *SLCO1B3* were lower in IM+ chickens than in IM− chickens (Fig. 2A), as were the expression levels of the OATP1B3 protein (Fig. 2B). The difference in the expression of *SLCO1B3* between IM+ and IM− chickens was also validated by the qPCR analysis (Fig. S1), the primers for which have been described in a previous study^25^. The expression levels of the *SLCO1B3* and OATP1B3 in IM+ chickens were both approximately 1/twofold compared with those in IM− chickens. Herein, we used IM+ Yimeng chickens as the *SLCO1B3* knockdown model to perform functional analysis in the liver using a gene enrichment method.

### Functional enrichment analysis of the DEGs

**Figure 3:**
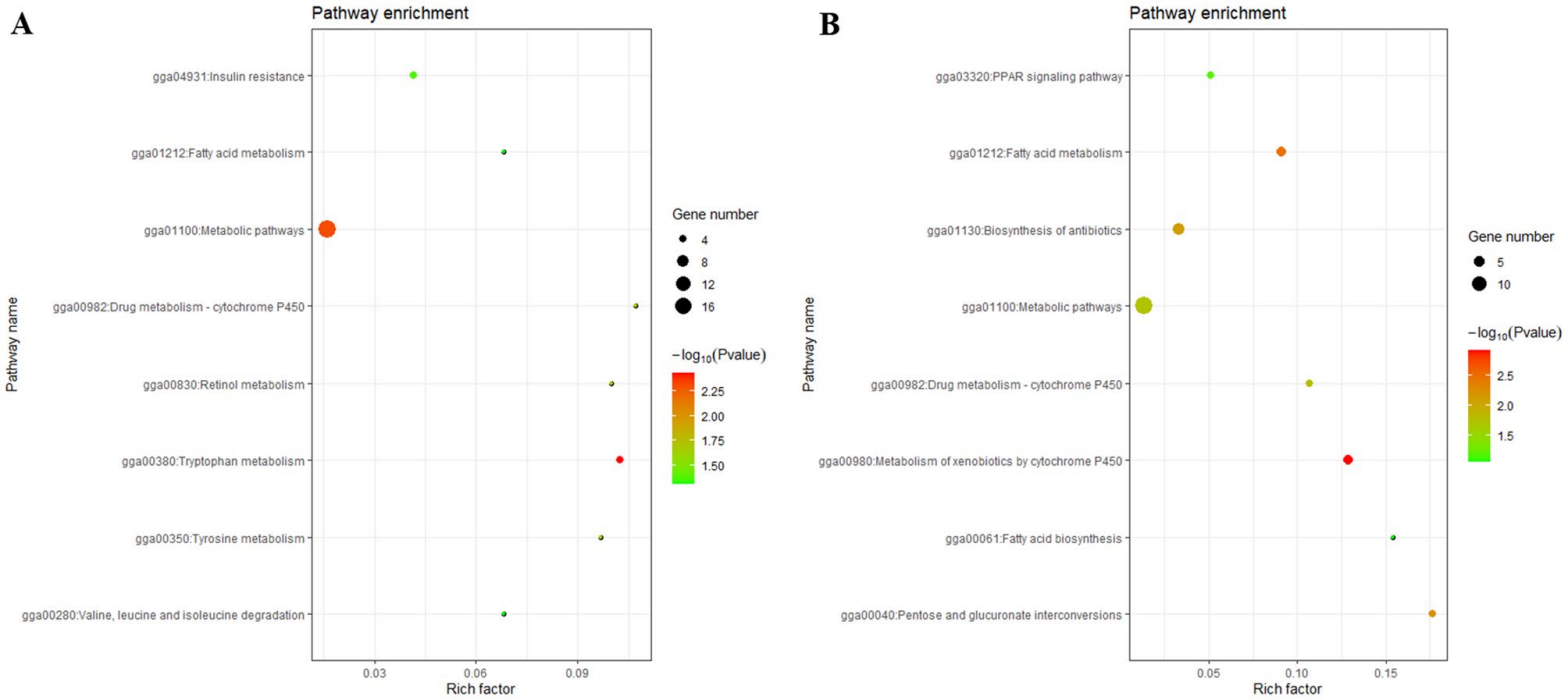
KEGG pathway analysis of the differentially expressed genes (**A**) and proteins (**B**). Fatty acid metabolism and exogenous substance metabolism related pathways can be significantly enriched.

The regulation network differences for the *SLCO1B3* between the IM+ and IM− hens were investigated. We performed a functional enrichment analysis of the DEGs using DAVAID 6.8 online software. The DEGs were significantly enriched in the pathways, including the tryptophan metabolism, metabolic pathways, drug metabolism—cytochrome P450, Retinol metabolism, Tyrosine metabolisminsulin resistance, and fatty acid metabolism (Fig. 3A). The main GO terms included iron ion binding, cell, response to antibiotic, NAD binding, and oxidative deethylation (Fig. S2A). There were also some DEGs that could be classified according to their gene symbol and function. The liver microsome CYP450 category for the DEGs included *CYP2C18*, *CYP1A1*, *CYP1A2*, and *CYP3A4,* which play an important role in xenobiotics and drug metabolism. The lipid biosynthesis DEG category included *SCD*, *FASN*, *ELOVL6*, *THRSP*, and *ME1*. In addition, some immune related genes, such as *NFKBIA*, *NFKBIZ* highly expressed in the IM+ chickens, *IL1R1L* highly expressed in the IM− chickens. We will try to discuss this phenomenon later.

### Functional enrichment and protein–protein interaction analysis of DEPs

**Figure 4:**
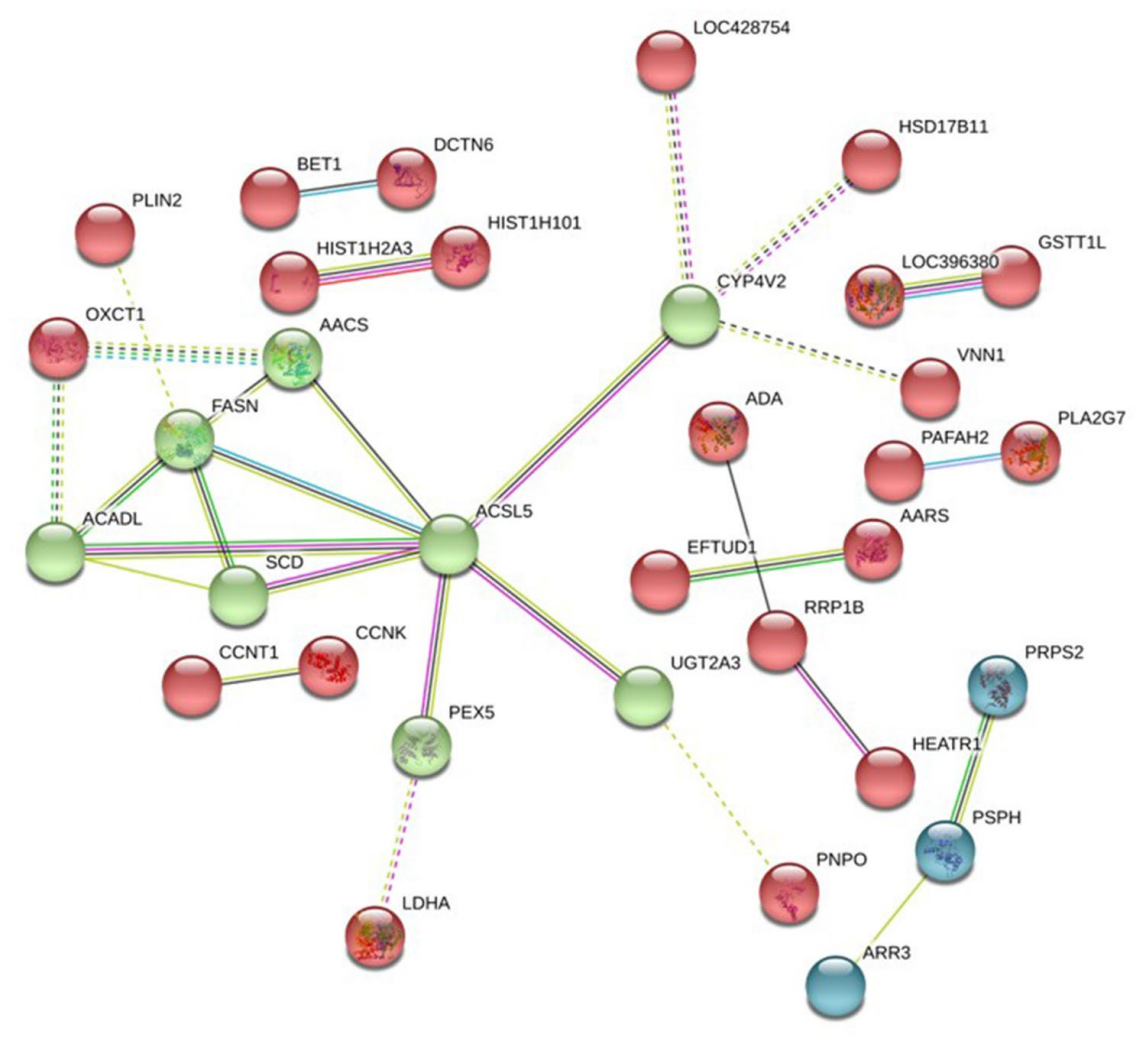
Protein–protein association networks of the differentially expressed proteins (DEPs). The green nodes can associate with each other and the majority proteins take part in the fatty acid metabolism. Disconnected nodes are hidden from the network. Colored nodes represent query proteins and first shell of interactors. Edges represent protein–protein associations, the detail legends can find in the String online database (https://string-db.org/).

Proteome enrichment analysis of the DEPs showed that the main KEGG pathways identified were the metabolism of xenobiotics by cytochrome P450, fatty acid metabolism, biosynthesis of antibiotics, and drug metabolism-cytochrome P450 (Fig. 3B). The main GO terms contained metabolic processes, fatty acid biosynthetic processes, and cytosol (Fig. S2B). The main fatty acid biosynthesis DEPs included SCD, FASN, ACSL5, ACADL, and the CYP450-drug or xenobiotic metabolism-related DEPs, including UGT2A1, LOC396380, DHDH, GSTT1L, and RP11-400G3.5 (CYP2C9-like). Several strong interactions were found among the DEPs (Fig. 4). The SCD, FASN, ACSL5, ACADL, AACS, CYP4V2, PEX5, and UGT2A1 proteins played pivotal roles in the interaction networks.

### Comparative analysis of the transcriptome and proteome

In the functional analysis, fatty acid metabolism and drug metabolism—cytochrome P450 were all significantly enriched in the two omics studies. Furthermore, some key genes involved in fatty acid metabolism, such as SCD and FASN in the proteome, had lower expression levels in IM+ chickens than in IM− chickens. The proteome analysis results validated the transcriptome analysis. We noted that some transcript factor genes in the RNA-seq were significantly different between the IM+ and IM− chickens, for example, *THRSP*, *AHR*, and *IGFBP1*, whereas they were not identified in the proteome analysis. This may be because the transcript factor protein content was exceptionally low in the tissues. These transcription factors could be the direct reason for the gene expression regulation of the fatty acids and exogenous substance metabolism.

### Differences in serum biochemical indexes between IM + and IM− hens

To reflect the difference of lipid metabolism between IM+ and IM− chickens, serum biochemical indexes of the two groups were detected. The IM+ and IM− Yimeng hens came from the same herd, the same period, the same feeding conditions. Six serum biochemical indexes including APOA1, APOB, TBA, HDL-C, LDL-C and VLDL-C, were measured to show the difference of lipid metabolism between IM+ and IM− chickens. Five of them showed significant difference between groups. Serum concentrations of HDL­C, LDL­C, and TBA in the IM+ birds were significantly lower than those in the IM− birds (*P* ≤ 0.05), and serum APOA1 and APOB concentrations in the IM+ birds were extreme significantly lower than those in the IM− birds (*P* ≤ 0.01), while no significant difference was detected in VLDL-C (Table S3).

### *EAV-HP* transcript identified and full-length open reading frame cloned

**Figure 5:**
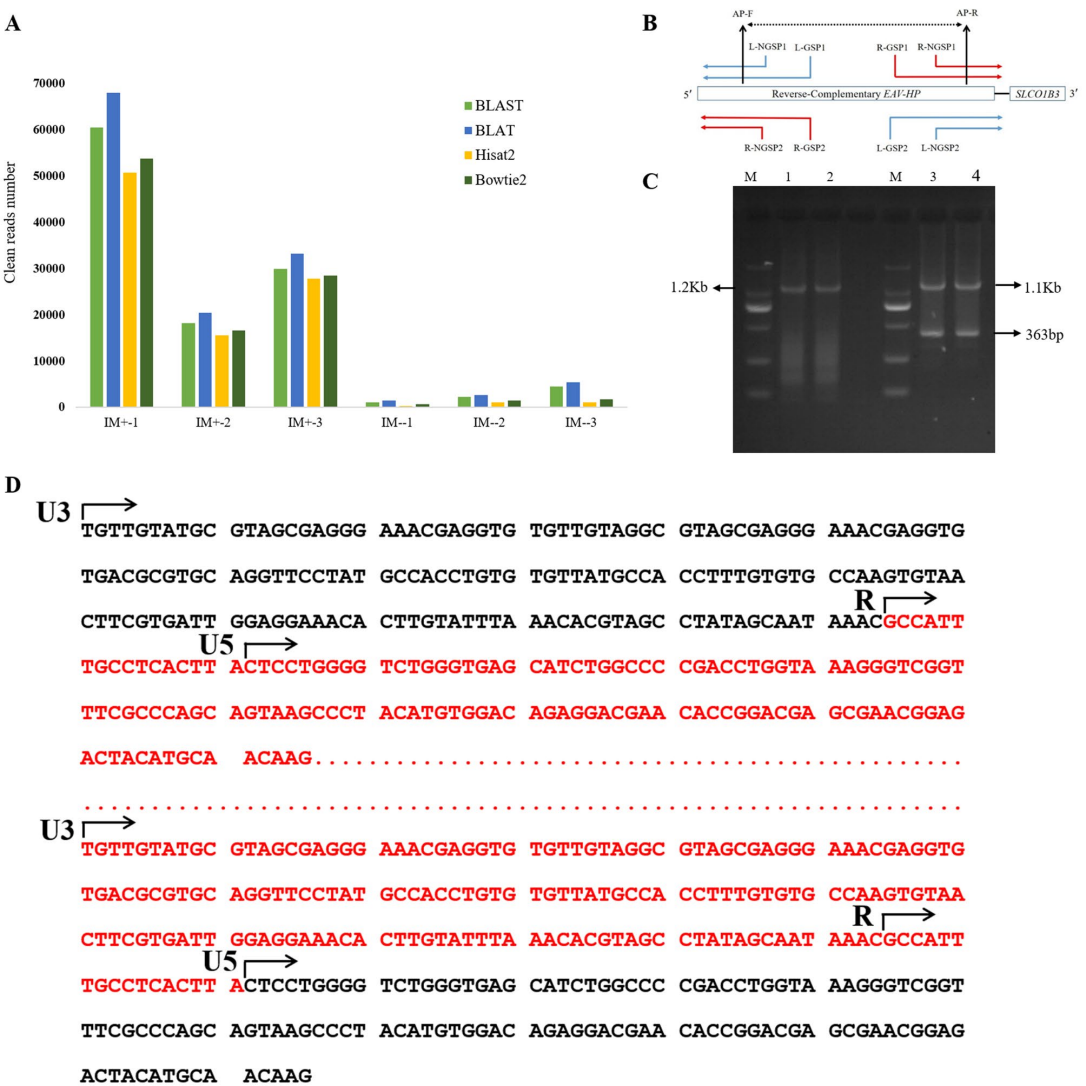
A new *EAV-HP* transcript highly expressed in the liver tissues of IM+ chickens. (**A**) *EAV-HP* alignment results from the RNA-seq data. y-axis represents the mapped clean read number for each individual library. BLAST, BLAT, Hisat2 and Bowtie2 are four different R packages used to align the *EAV-HP* sequence. The different software packages have different features and were used to obtain consistent results. **(B**) The *EAV-HP* transcript RACE primers design model. The blue colored RACE primers were designed to clone the *EAV-HP* transcript transcription start site from the left orientation, and the red color from the right orientation. (**C**) RACE gel results of the *EAV-HP* transcript, the gel cropped from same part of the same gel and the full-length gel are given in the Fig. S4, 1, 2 indicate the R-5ʹRACE nested PCR results; 3, 4 indicate R-3ʹ RACE nested PCR results. The short fragment (363 bp) for 3ʹ RACE was a false positive from the later sanger sequencing. (**D**) The transcriptional start and end site of the *EAV-HP* transcript. The red colored sequence is the full-length *EAV-HP* transcript sequence, and the red dots represents the omitted gag and env sequences, U3, R and U5 are the parts of long terminal region (LTR) of *EAV-HP*.

We noticed that there is one transcript (*LOC107051636*) significantly differentially expressed between IM+ and IM− chickens in the RNA-seq, the fold change was up to 52 times. After sequence alignment, we found that the transcript had 84.77% similarity to the *EAV-HP* fragment (GeneBank: JF837512). We then compared the clean reads number of transcriptome sequencing with the *EAV-HP* sequence, we found that the comparable reads of IM+ group were all more than 10,000 reads, while the reads number in IM– group were very low (Fig. 5A). We speculated that the differential expression of *EAV-HP* transcript has relationship to the *EAV-HP* IM+ in the upstream of *SLCO1B3*. After comparison in the database, we found that there were 15 specific SNPs between the *EAV-HP* IM+ and other *EAV-HP* sequence in the genome (Table S4). Therefore, we calculated the number of reads with the above specific SNPs, the results showed that more than 95% of the mapped reads in each SNP locus in the IM+ individuals contained special SNPs, while no specific SNP was contained in reads of IM− individuals (Table S5). This indicated that the *EAV-HP* transcript highly expressed in the liver of IM+ chickens come from the *EAV-HP* insertion in the 5*ʹ* region of *SLCO1B3*.

To ensure the *EAV-HP* transcript transcriptional start sites and orientation, two orientation (left and right, shown in Fig. 5B) 5ʹ and 3ʹ RACE experiments were performed, the results showed that the left RACE did not clone any fragment, while the right direction RACE successfully amplified specific fragments (Fig. 5C), suggesting that the *EAV-HP* transcription starts in the reverse orientation. The full-length *EAV-HP* transcript sequence was also cloned, which is a new *EAV-HP* transcript starting from the R region of the long terminal region (LTR) and ended in another R region of the LTR (Fig. 5D), having a complete open reading frame (Fig. S3).

### qPCR verification of DEGs

To confirm the repeatability and accuracy of the RNA-seq gene expression data obtained from the chicken liver libraries, qPCR was carried out on 9 selected DEGs: transporter genes *SLC16A1*, transcription factor genes (*THRSP*, *IGFBP1*, *AHR*), antibacterial peptide gene (*LEAP2*), fatty acid synthesis gene (*ELOVL6*), inflammatory factor gene (*NFBKBZ*), microsome CYP450 representative gene (*CYP2C18*), and the *EAV-HP* transcript were also selected for gene expression validation. The qPCR analysis results matched well with the RNA-seq results (Table 2), indicating that the RNA-seq analysis results are reliable. The correlation coefficient between the qPCR and RNA-seq was 0.997 (*P* < 0.001), indicating good reproducibility of the differential expression results obtained by the RNA-seq.

**Table 2:** Expression patterns of the 11 mRNAs selected for qPCR validation.

Gene symbol	qPCR		RNA-seq	
	Fold change	*P* value	Fold change	*P*_*adj*_ value
*LEAP2*	0.22	0.0013	0.16	0.0060
*AHR*	2.77	0.0158	2.02	0.0235
*ELOVL6*	0.42	0.0487	0.35	0.0108
*THRSP*	0.07	0.0032	0.06	0.0060
*IGFBP1*	6.96	0.0047	6.19	0.0060
*NFKBIZ*	3.13	0.0055	2.21	0.0060
*SLC16A1*	3.14	0.0030	1.82	0.1236
*CYP2C18*	0.15	0.0396	0.15	0.0060
*EAV-HP*	96.38	0.0053	51.40	0.0060

## Discussion

In humans, *SLCO1B3* is mainly expressed in the liver and its encoded protein OATP1B3, plays an important role in endogenous and exogenous transport processes, which has a closely relationship with bile acids and drug metabolism in liver^3,20^. Our previous research also showed that chicken *SLCO1B3* was mainly expressed in liver, and the *SLCO1B3* homolog gene *SLCO1B1* had exceptionally low expression levels in the liver^25^. This may indicate that in chickens, *SLCO1B3* plays more important role in the endogenous and exogenous transport processes in the liver. On one hand, the *EAV-HP* insertion upstream of *SLCO1B3* gene resulted in the chicken blue eggshell phenotype, on the other hand, it also reduced the expression level in liver. In this study, we compared the liver transcriptome and proteome of IM+ and IM− groups. These two groups come from the same population, under the same feeding environment, and have high similarity in genetic and environmental background, which means that the differentially expressed genes and pathways found in the transcriptome and proteome are more related to the variation and down-regulation of *SLCO1B3* gene. So here we used the IM+ hens as *SLCO1B3* gene knockdown animal models to investigate the function of this gene.

Both transcriptome and proteome analysis confirmed the enrichment of fatty acid metabolism pathway. Comparison with IM− group, the expression level of genes in this pathway was mainly significantly decreased in IM+ group. *THRSP* can regulate *FASN*, *ACL*, and *ME1* expression after activation by the thyroid hormone^27^. Estradiol-17β glucuronide and estrone-3-sulfate are the most important estrogens in vertebrate animals, and could be transported by OATP1B3, which may control fatty acid metabolism in livers of laying hens^28–31^. In conclusion, we speculated that *SLCO1B3* could regulate liver fatty acid metabolism by transporting thyroid hormones, estrogens, and bile acids indirectly, which are all ligands of OATP1B3^3,11,32^. Unlike mammals, more than 70% of bird fatty acid synthesis occurs in the liver^33^. The liver fatty acid metabolism was lower in IM+ hens and was also reflected in the serum biochemical parameters. The apolipoprotein cholesterol in the serum could affect the accumulation of nutrients in the egg yolk, especially the cholesterol^34^. The nutrient content of the IM+ Yimeng chicken eggs should be investigated further in future investigations.

It was also noted that the metabolism of xenobiotics and drugs by the cytochrome P450 pathway was enriched in the RAN-Seq and proteome analyses. Transcriptome analysis revealed several DEGs categories, including the liver microsome CYP450s. CYP450s play a very important role in the metabolism of arachidonic acid, prostaglandins, retinol, retinoic acid, and cholecalciferol^35^. Some studies have summarized the function of liver microsome CYP450s in the metabolism of drugs in humans^36^. The nuclear receptors AHR can regulate the expression of the liver microsome CYP450s either continuously or when induced^37,38^, and some ligands, including various endogenous and xenobiotic substrates, can be transported by OATP1B3. OATP1B3 may thus regulate the metabolism of xenobiotics by controlling the activity of CYP450s^18,19,39,40^ The results of the present study are consistent with previous reports about the involvement of human *SLCO1B3* in the metabolism of xenobiotics in the liver^15,16,41^. From this perspective, the chickens with blue eggshells could function as a liver *SLCO1B3* gene knockdown model for drug and toxic metabolism experiments in the future.

**Figure 6:**
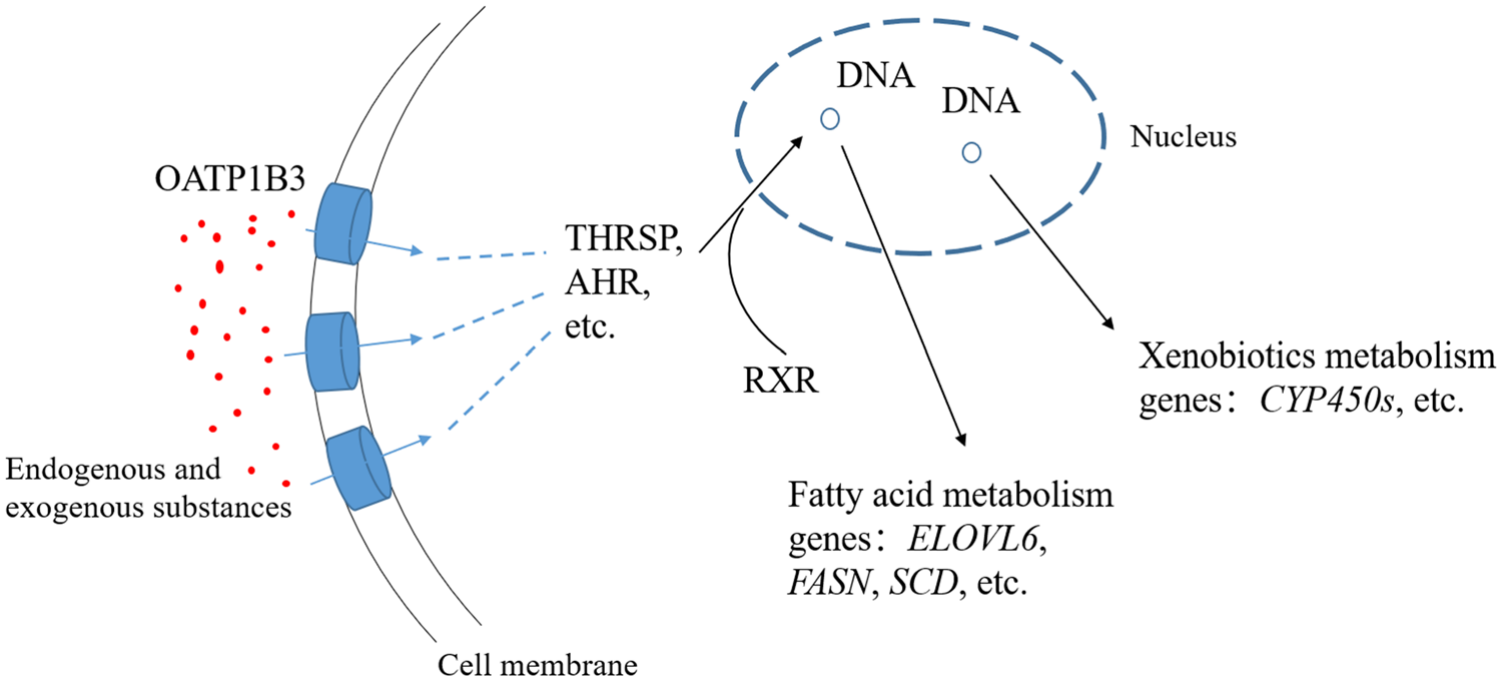
Chicken *SLCO1B3* gene functional regulatory model in the liver. THRSP, AHR, FXR, PPAR represents transcription factors and RXR are their nuclear receptor, they can bind each other and form heterodimer. Many endogenous and exogenous substances are the ligands of the heterodimers descript above. We speculate that the OATP1B3 can regulate the cell fatty acid metabolism and xenobiotics metabolism via transporting various endogenous and exogenous substances.

Herein, we propose a schematic diagram of the function of the chicken *SLCO1B3* gene in the liver tissue (Fig. 6), by combining the enrichment analysis of the RNA-seq and proteome with the gene function of human *SLCO1B3*. Chicken OATP1B3 protein may regulate the activity of ligands-dependent on transcription factors, to regulate the expression of genes related to fatty acid metabolism and exogenous substance metabolism, by controlling the amount that endogenous and exogenous substances are transferred (including bile acids, thyroid hormone, estrogens, etc.) into the liver cells. Although some transcription factors (e. g., ESR and PPAR) were not significantly expressed or detected in the RNA-seq and proteome.

It was reported that IR-1 is an important element which can regulated the human *SLCO1B3* gene expression in the liver^17^. Our LMH cells experiment analysis revealed that there is a predicted IR-1 like element in the 5ʹflanking region of *SLCO1B3*, which also can be activated by the bile acids. It helped us to understand why *EAV-HP* insertion decreased the expression of *SLCO1B3* in the liver. Furthermore, a chicken specific bile acid activated element IR-1 like has been cloned and validated in this study, it will be helpful to study the expression of cholate responsive genes in the future.

Endogenous retroviruses widely exist in vertebrate genomes. *EAV-HP* is a subtype of chicken endogenous virus and has been investigated for almost 30 years and is commonly found in avian species^42^. It is widely accepted that Avian Leukosis Virus-J (ALV-J) originated from the recombination of *EAV-HP* and an ancient ALV^43^. *EAV-HP* expression occurs during the embryonic period^44^, and with an immune tolerance when hatched meat-type chickens are infected with ALV-J^43^. Since its emergence, ALV-J has caused high levels of economic loss. However, whether the expression of *EAV-HP* can induce chicken immunity has not been reported. In the study of human endogenous retrovirus, it was found that endogenous retrovirus was related to human innate immune response^45–47^, which should arouse our attention to the relationship between endogenous retrovirus and immunity.

It is widely accepted that most endogenous retroviral elements are silenced in the genome, however, there are still some reports showed that some subtypes of retroviruses are expressed in embryonic stage in humans^47^. Here the *EAV-HP* transcript was identified in the IM+ group, while it was barely detected in the IM− group with RNA-seq and qPCR analyses. There are at least seven almost complete *EAV-HP* sequences identified in the most recent chicken reference database (Table S4), but only the *EAV-HP* insertion in the promoter region of *SLCO1B3* was highly expressed. Promoters of the chicken *SLCO1B3* have been discussed in a previous investigation^25^. However, the *EAV-HP* transcript RACE analysis in this study showed that its high expression levels may not be due to the promoter of the chicken *SLCO1B3* gene, but a detailed investigation is required to better understand this. *EAV HP* transcripts have a complete open reading frame (Fig. S3), which means that some viral protein elements (env, gag) can be translated. These protein fragments may play the role of antigen and cause immune response. The biosynthesis of antibiotics pathways and the regulation of inflammatory response terms were identified in our omic analysis, as some immune genes, such as *NFKBIZ*, *NFKBIA*, and *IL1RL1*, and some proteins such as PPIA, that function to inhibit viruses^48,49^ was differentially expressed between two groups. However, the comprehensive effects of the *EAV-HP* transcripts on the chicken immune system, its health and some potential roles in the new virus formation require further investigation.

In conclusion, we cloned a chicken cholate responsive element in the 5ʹ flanking region of *SLCO1B3*, which is helpful to understand the expression of *SLCO1B3* in IM+ individuals, and is conducive to the future research of chicken bile salt response genes. We provided new insights into the functions of *SLCO1B3* in the metabolism of lipids and xenobiotics in chickens by comparing the Transcription and proteomic expression between IM+ and IM− individuals. *EAV-HP* insertion greatly decreased the expression of *SLCO1B3* and activated itself expression at the same time. The high expression of *EAV-HP* is firstly reported here, which may induce liver immune response and need further study. Our study provided another example of pleiotropic variation in domestic animals, it might be useful in the current breeding of the blue eggshell chickens.

## Methods

### Animals and sample preparation

**Figure 7:**
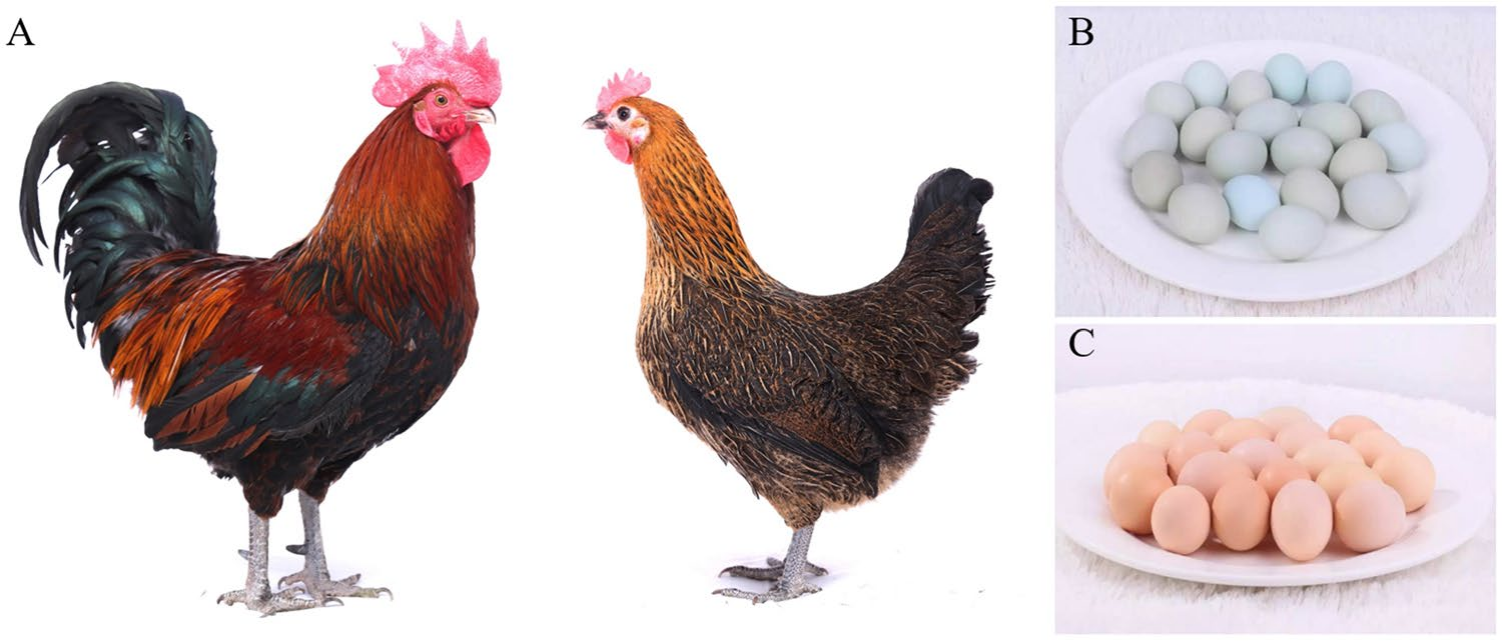
(**A**) Yimeng rooster (left) and hen (right). (**B**) The chicken with blue-green-shelled eggs can represent the presence of *EAV-HP* inserted mutation (IM+) in the 5′ region of *SLCO1B3*. (**C**) The chicken with brown-shelled eggs can represent without *EAV-HP* insertion (IM−).

Yimeng chickens (Fig. 7A) was separated into IM+ or IM− groups, which can be selected based on the eggshell color (Fig. 7B C), were obtained from Shandong Longsheng Agriculture and Animal Husbandry Group Co., Ltd., Linyi City, Shandong Province, China, for use in this study. In this case, the IM+ chickens were all heterozygotes, with one copy of the *EAV-HP* insertion, while the IM− chickens were all recessive homozygotes. Six hens (n = 3 IM+ and n = 3 IM−) were slaughtered at the same age (approximately 300 days). Furthermore, 2 mL whole blood samples were collected from the wing veins of 42 chickens (21 IM+ chickens and 21 IM− chickens) without providing an anticoagulant. Serum was separated from the whole blood samples by centrifugation at 3000×*g* for 10 min at room temperature, and then stored in 1.5 mL centrifuge tubes at − 20 °C, for the biochemical analysis. The liver tissues were divided into two and immediately snap-frozen in liquid nitrogen and stored at − 80 °C, for later analysis. The molecular identification method of the IM insert mutation in Yimeng chickens was performed according to a previously described multiple PCR method^21^.

All procedures and experiments performed in this study were approved by the Animal Care and Use Committee of China Agricultural University (Approval no. XK257), all methods were performed in accordance with ARRIVE guidelines (https://arriveguidelines.org), and all methods were carried out in accordance with relevant guidelines and regulations.

### Vector construction, cell culture, transient transfection and dual luciferase assay

About 2000 bp (IM−) and ~ 4.8 Kb (IM+ , including 4.2 Kb *EAV-HP* insertion) upstream region from the transcription start site of the *SLCO1B3* was cloned by Taq DNA polymerase and ligated into the pMD19-T vector (Takara Biomedical Technology (Beijing) Co., Ltd, Beijing, China) as per the manufacturer’s guidelines. After sequencing by a Sanger method (Sino Geno Max, Beijing, China), seven different length fragments of the upstream of *SLCO1B3* was cloned using PCR with primers contained specific restriction site listed in Table S6, then they were inserted into the multiple copy region of pGL3-Basic vector (Promega, Beijing, China). A chemical synthesis method was used to the mutated vector construction (Beijing Genomics Institute, Beijing, China). Constructed plasmids were extracted using the plasmid midi kit (Tiangen Biotech Co., Ltd, Beijing, China) as per the manufacturer’s guidelines. Chenodeoxycholic acid (CDCA) (Sigma-Aldrich, MO, USA) dissolved in DMSO into a storage solution of 100 mM. LMH cells were cultured in Waymouth’s medium plus 10% fetal bovine serum, 1% penicillin/streptomycin and different concentration CDCA. All reagents for cell culture were purchased from Invitrogen/Gibco. Cells were seeded into 24-cell plates (Corning, NY, USA) and were co-transfected with 180 ng pGL3-construct and 20 ng pRL-TK using Lipofectamine 2000 (Invitrogen, CA, USA) as per the manufacturer’s guidelines. Each construct has three replicates, including pGL3-Basci vector. After 36 h, firefly and renilla luciferase activities were measured using the Dual-Glo Luciferase Assay System (Promega) as per the manufacturer’s guidelines.

### RNA-seq library preparation and sequencing

Total RNA was extracted from the liver using an RNA prep Pure Kit (Tiangen) as per the manufacturer’s guidelines. Six mRNA libraries were constructed and RNA degradation and contamination were visualized on 1% agarose gels. RNA purity was checked using a NanoPhotometer spectrophotometer (Implen, CA, USA), and concentrations were determined using a Nanodrop 2000 (Life Technologies, CA, USA). RNA integrity was assessed using the RNA Nano 6000 Assay Kit for the Bioanalyzer 2100 system (Agilent Technologies, CA, USA) as per the manufacturer’s guidelines. A total of 3 μg RNA from each sample was used as the input material for RNA sample preparations. The mRNA sequence libraries were constructed using a NEBNext Ultra Directional RNA Library Prep Kit for Illumina (New England Biolabs, Inc., MA, USA), according to the manufacturer’s recommendations. Subsequently, the mRNA libraries were sequenced on an Illumina Hiseq 2500 platform, and 100-bp paired-end reads were generated.

### Read mapping

The clean reads were obtained from the raw reads by removing the reads containing adapters, poly-N regions, and low-quality reads. The Q20 scores and Q30 scores of the clean data were calculated. The clean reads were mapped to the chicken genome assembly (galGal6), which was downloaded from the Ensembl database. The reference genome index was constructed using Bowtie v2.0.6, and the clean paired-end reads were aligned to the reference genome using TopHat v2.0.9^50,51^. The mapped reads of each sample were assembled using Cufflinks (v2.1.1)^52^ with a reference-based approach. The Cuffdiff (v2.1.1) algorithm was used to calculate the fragments per kilobase of exon per million mapped fragments (FPKMs) of the coding genes in each sample, using a model based on the negative binomial distribution^53^. All RNA-Seq RAW data were deposited in the SRA database under accession number PRJNA680850, link is (https://www.ncbi.nlm.nih.gov/sra/PRJNA680850).

### Protein extraction, reduction, alkylation, digestion, TMT labeling and LC–MS/MS

Protein extraction was carried out according to a previously described method^54^, and the final protein concentration was quantified using the BCA (Beyotime Biotechnology, Beijing, China) assay as per the manufacturer’s guidelines. The proteins’ disulfide bonds of the samples were broken by mixing with a solution of 10 mM DTT for 1 h. Thereafter, these samples were incubated in 50 mM iodoacetamide for alkylation at room temperature for 1 h away from light. Trypsin was used for protein digestion at a volume ratio of 1:50 at 37 °C for 14 h and 1 μL of formic acid was added to the solution for stopping the enzymatic digestion. Finally, peptides were concentrated using a SpeedVac system (Marin Christ, Osterod, Germany). The sample peptide was labeled with TMT kits (Thermo Fisher Scientific, USA) as per the manufacturer’s guidelines. The samples were then combined and stored at -80 °C until the following LC/MS analysis. the peptide sample was loaded onto an LC–MS system. The peptide enrichment, eluted, MS/MS data collected and saved according to a previously study^54^. Data are available via ProteomeXchange with identifier accession PXD022768.

### Database search, protein identification, and quantification

The MS/MS data were searched against a NCBI RefSeq and Ensembl combined database (txid9031Gallus-gallus32176. Fasta, 32,176 entries, Gallus_gallus.Gallus_gallus-5.0.pep.all.fa.gz) for the peptide identification and quantification using Mascot 2.1 and Proteome Discoverer1.4 software (Thermo Fisher Scientific). The parameters were set as follows: Search parameters were trypsin specificity, carbamidomethyl as a fixed modification, oxidation and phosphorylation as variable modifications, with two allowed missed cleavages per peptide; three maximum allowed variable PTM per peptide Precursor mass tolerance was set at 15 ppm, and fragment ion tolerance at 0.02 Da. Protein identifications were only considered confident if at least two unique peptides with at least two spectra were identified. The protein expression profile was done by hierarchical clustering to create an expressional profile of differentially expressed protein groups between IM+ and IM− chickens.

### Measurement of serum biochemical parameters

Six serum biochemical parameters were assessed using kits, including a high-density lipoprotein cholesterol (HDL-C) kit, low-density lipoprotein cholesterol (LDL-C) kit, apolipoprotein A-I (APOAI) kit, and apolipoprotein B (APOB) kit were purchased from Biosino Bio-Technology and Science Inc. (Beijing, China). Furthermore. a total bile acid kit was purchased from Zhejiang Weiyi Bio-tech. Co., Ltd. (Zhejiang, China) and a very low-density lipoprotein cholesterol (VLDL) kit was purchased from the Beijing Sino-UK Institute of Biology Technology (Beijing, China). These parameters were measured by following the manufacturer’s instructions for all kits using a Mindray IM–420 fully automated clinical chemistry analyzer (Mindray Bio-Medical Electronics Co., Ltd., Shenzhen, China) at the Beijing Sino-UK Institute of Biology Technology (Beijing, China).

### Cloning the full-length *EAV-HP* transcript

The IM+ Yimeng chicken liver tissue RNA for RNA-seq was used for the 5ʹRACE and 3ʹRACE experiments. The RACE experiments followed the protocol of SMARTer RACE 5ʹ/3ʹ Kit (Clontech, Mountain View, CA, USA). Gene RACE primers (including nested primers) were designed for the two orientations as the transcriptional start site for the *EAV-HP* could not be confirmed. In addition, another primer (AP) set was designed for the entire cDNA sequence of the *EAV-HP*, as the products of the corresponding RACE primer sets could not complete the entire sequence. The primer sequences are presented in Table S7, and the PCR products were sequenced using the Sanger method (Sino Geno Max).

### Quantitative real-time PCR

cDNA was synthesized from 1.5 μg of the extracted total RNA (RNA-seq samples) using the Fast King RT Kit (Tiangen) as per the manufacturer’s guidelines. The qPCR was performed using a previously descript method^25^. The glyceraldehyde-3-phosphate dehydrogenase (*GAPDH*) was chosen as the house keeping gene to correct gene expression, and all the qPCR gene-specific primers were designed using Primer Premier 5.0 software. The primer sequences are presented in Table S8.

### Bioinformatics and statistical analysis

DAVID 6.8 (https://david.ncifcrf.gov/)^55,56^ online software was used to perform the GO annotation and KEGG pathway analysis. STRING v11 (https://string-db.org/) online software was used for the protein–protein interaction analysis. The entire *EAV-HP* fragment sequence was downloaded online, which was submitted by Wang et al. 2013 (accession number: JF837512)^21^. Four R packages: BLAST^57^, BLAT^58^, Hisat2^59^, and Bowtie2^60^ were used to align the *EAV-HP* sequence, using a clean read mapping method. The parameters of the alignment software were set to their defaults. T-tests were performed to determine significant differences in the serum biochemical parameters between the IM+ and IM− chickens, using SAS 9.2 software, with differences being considered significant at *P* ≤ 0.05.

## Supplementary Information

Supplementary Information 1.

Supplementary Information 2.

Supplementary Information 3.

Supplementary Information 4.

Supplementary Information 5.

Supplementary Information 6.

Supplementary Information 7.

Supplementary Information 8.

Supplementary Information 9.
