# Supplementary material for: An EAV-HP insertion in the promoter region of SLCO1B3 has pleiotropic effects on chicken liver metabolism based on the transcriptome and proteome analysis
Source: Sci Rep. 2021 Apr 7;11:7571. doi: 10.1038/s41598-021-87054-9 (PMC8026973; doi:10.1038/s41598-021-87054-9)
Supplement: Supplementary file 1 — Supplementary Information 1. [file 41598_2021_87054_MOESM1_ESM.docx]

**An *EAV-HP* insertion in the promoter region of *SLCO1B3* has pleiotropic effects on chicken liver metabolism based on the transcriptome and proteome analysis**

Jianfei Chen^1^, Guoying Hua^1^, Deping Han^2^, Xiaotong Zheng^1^, Xianggui Dong^1^, Shuxiang Wang^1^, Junjiang Long^3^, Zhonghua Zheng^3^, Ailing Wang^3^, Jiankui Wang^1^, Xiaotong Wang^4^, and Xuemei Deng^1*^

^1^Key Laboratory of Animal Genetics, Breeding and Reproduction of the Ministry of Agriculture & Beijing Key Laboratory of Animal Genetic Improvement, China Agricultural University, Beijing 100193, China

^2^College of Veterinary Medicine, China Agricultural University, Beijing 100193, China

^3^Shandong Longsheng Agriculture and Animal Husbandry Group Co., Ltd., Linyi 276000, China

^4^School of Agriculture, Ludong University, Yantai 264000, China

^*^Corresponding to: deng@cau.edu.cn

**Supplementary information**

[**Supplementary Figures: 2**](#_Toc64644594)

[**Supplementary Figure S1 2**](#_Toc64644595)

[**Supplementary Figure S2 2**](#_Toc64644596)

[**Supplementary Figure S3 3**](#_Toc64644597)

[**Supplementary Figure S4 3**](#_Toc64644598)

[**Supplementary Tables: 4**](#_Toc64644599)

[**Supplementary Table S1: Please see the Excel file 4**](#_Toc64644600)

[**Supplementary Table S2: Please see the Excel file 4**](#_Toc64644601)

[**Supplementary Table S3: Please see the Excel file 4**](#_Toc64644602)

[**Supplementary Table S4: Please see the Excel file 4**](#_Toc64644603)

[**Supplementary Table S5: Please see the Excel file 4**](#_Toc64644604)

[**Supplementary Table S6: Please see the Excel file 4**](#_Toc64644605)

[**Supplementary Table S7: Please see the Excel file 5**](#_Toc64644606)

[**Supplementary Table S8: Please see the Excel file 5**](#_Toc64644607)

# Supplementary Figures:

## **Supplementary Figure S1**


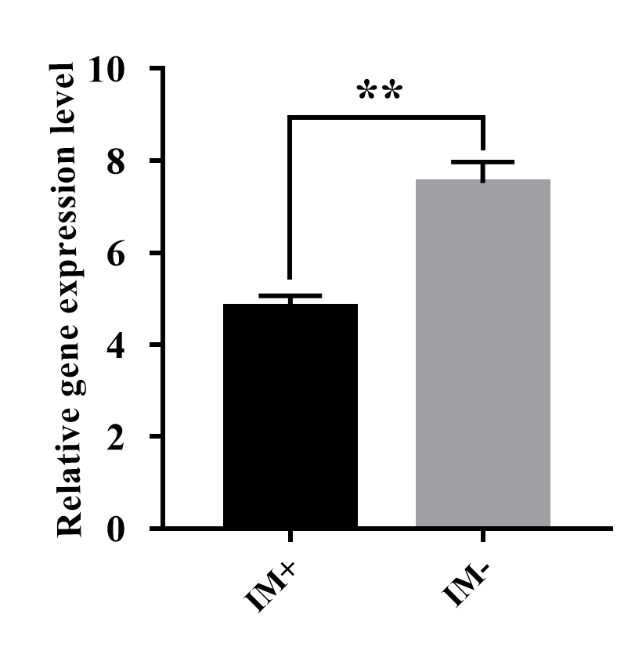


**Figure S1.** Relative *SLCO1B3* gene expression by q-PCR analysis.

## **Supplementary Figure S2**


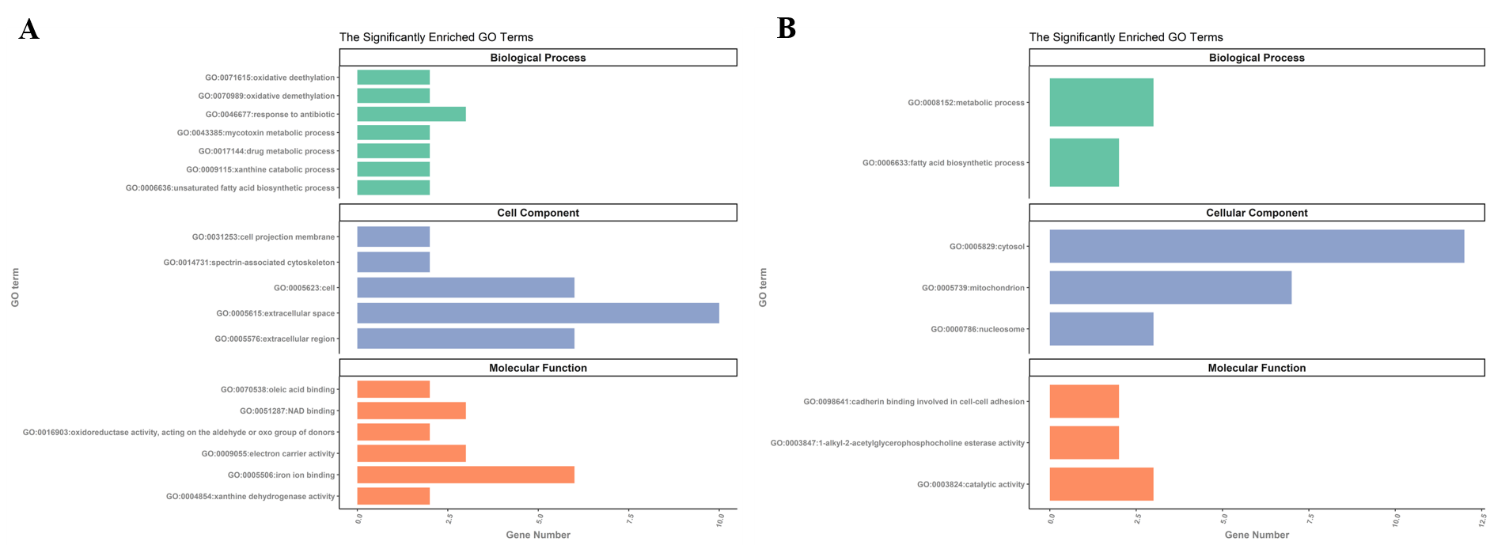


**Figure S2.** GO enrichment terms of differentially expressed genes (**A**) and proteins (**B**).

## Supplementary Figure S3


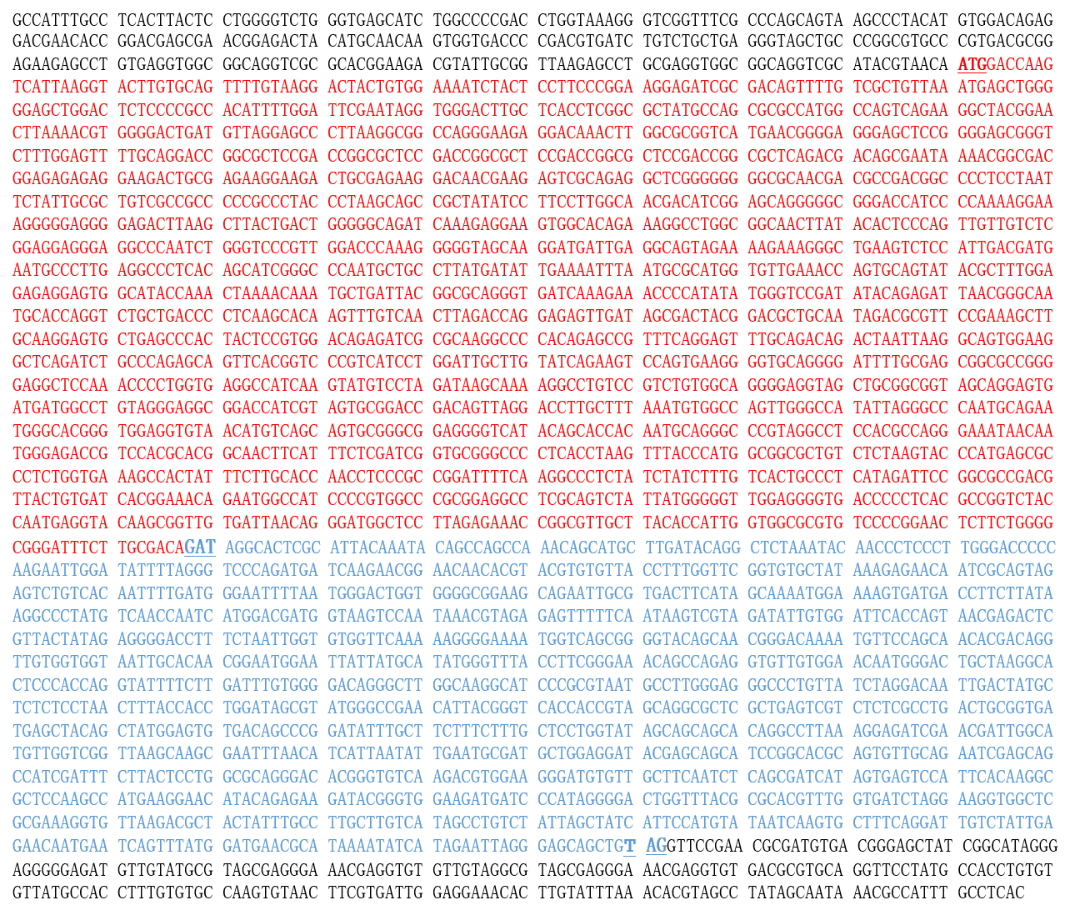


**Figure S3.** The compete *EAV-HP* transcript. The black region is the untranslated sequence of 5' and 3', the red region is the gag sequence, and the blue region is the env sequence. The underline indicates the start codon, the first amino acid of env and the termination codon.

## **Supplementary Figure S4**


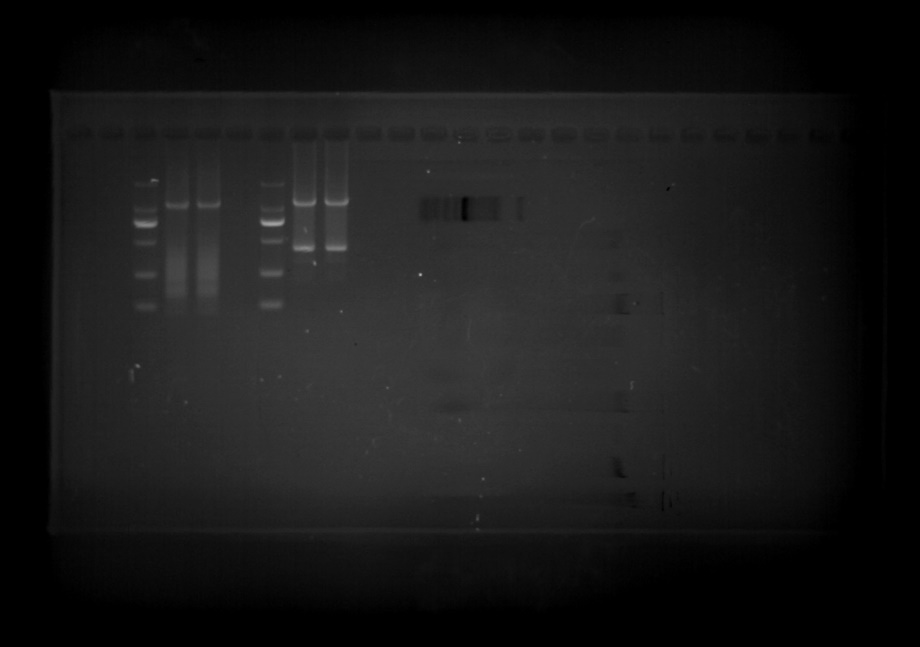


**A**


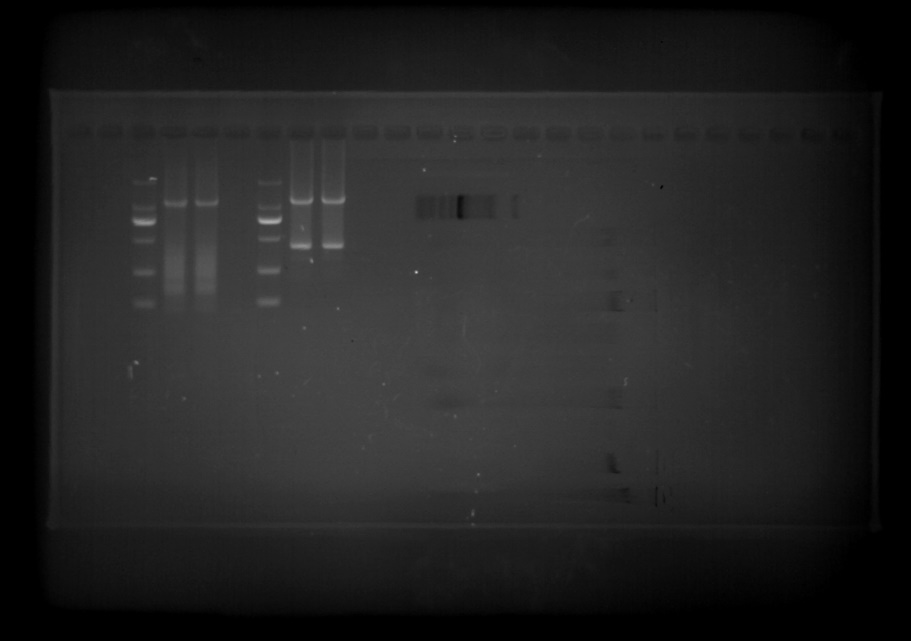


**B**

**Figure S4.** Full-length gels of RACE results with different exposures. (**A)** The original picture of Figure 5C. (**B**) Relative overexposure picture of Figure 5C.

# Supplementary Tables:

**Supplementary Table S1: Please see the Excel file**

**Table S1.** Differentially expressed genes list of RNA-seq.

**Supplementary Table S2: Please see the Excel file**

**Table S2.** Differentially expressed proteins list of proteomic.

**Supplementary Table S3: Please see the Excel file**

**Table S3.** Serum biochemical parameters test results of blue-green-shelled and brown-shelled Yimeng hens.

**Supplementary Table S4: Please see the Excel file**

**Table S4.** 7 relatively complete *EAV-HP* sequences founded in the latest chicken reference genome version using the BLAT of UCSC online database.

**Supplementary Table S5: Please see the Excel file**

**Table S5.** The reads number of each *EAV-HP* unique SNPs in each sample.

**Supplementary Table S6: Please see the Excel file**

**Table S6.** Primers used for fragment cloning.

**Supplementary Table S7: Please see the Excel file**

**Table S7.** PCR primers for *EAV-HP* RACE experiments.

**Supplementary Table S8: Please see the Excel file**

**Table S8.** PCR primers for quantitative real-time PCR validation of differentially expressed genes.
